# Proceedings of the Boston Organon Society

**Published:** 1888-07

**Authors:** S. A. Kimball

**Affiliations:** Secretary


### PROCEEDINGS OF THE BOSTON ORGANON SOCIETY.

Regular meeting of Organon Society held May 3d.

Dr. Wesselhœft read from the *Organon*, beginning at the 26th section.

Dr. Bell—In regard to the 26th section, I never quite understood or grasped the idea that the action of the remedy produced in the organism a stronger action than that of the disease. Of course, it is only a hypothesis, but it does not seem to me to be right.

Dr. Davis—Does it mean a stronger or a more potent effect?

Dr. Bell—It means a disturbance of the vital force. Now it does not seem necessary to produce a stronger effect on the vital force. I think the curative action of the remedy, and its power to cure, is a fact, and must be known as that. But the manner in which it is done must ever remain a mystery. All hypotheses on the subject only lead to argument without deciding anything.

Dr. Wesselhœft—I think he means that a weaker dynamic affection is permanently extinguished by a stronger dynamic affection.

Dr. Bell—In regard to the last clause of foot-note 14, "nor are they dependent on material morbific matter," I would add, "nor are they dependent on microbes."

Dr. Wesselhœft—The 28th section shows how Hahnemann regarded this explanation of the action of the remedy. He says in effect, accept it if you want to; but he says, "I therefore place but a slight value upon an attempt at explanation." These are all theoretical explanations and we will read them, but to me they have never been satisfactory. I believe that the indicated remedy acts antipathically, that it acts *against* the process of disease.

If we let the totality of the symptoms be represented by a straight line, and if we let the *exact* simillimum be represented by another straight line, when these two forces meet there is a cessation if they are of equal strength. A sudden stoppage of all symptoms and the patient recovers in twenty-four or forty-eight hours from what usually takes a week or two. I think we have all seen such cases where the exact simillimum has been found, and the result has been a sudden stoppage of disease action.

Instead of a sudden stoppage of action we may have oscillations backward and forward with the symptoms growing less and less, until they finally cease.

It is very important in such cases not to interrupt the action of the remedy. Then again we may not get the simillimum, but we get as nearly a simillimum as possible, the nearest remedy that we can find, a *satis simile*. This does not meet the force of the symptoms exactly as the simillimum did, but meets it at an angle, which develops a new force, a new set of symptoms, the resultant of the other two forces.

Now we try to get a simillimum for this new force. If we do it stops; if we only get a *satis simile*, still another force, or new train of symptoms, is developed, and so we zigzag them into health.

When a young man I had an Apis case which made a deep impression upon me. The patient was a young woman, with mania during menstruation, or rather, instead of a flow she would have violent congestions to the head with delirium. At these times she could not bear the slightest heat from fire or stove, and when exposed to it would have to go to the window or out-of-doors. I had given several remedies without relief, and it was just at this time that the proving of Apis was published. In reading it over I was struck with its resemblance to my case. I gave it, and the next menstruation was perfectly normal. When in Philadelphia some time after I related the case to Dr. Hering, and asked him what we would have done in this case without Apis. Dr. Lippe, who was present, said, "We would have zigzagged her into health by Sulph., Puls., and Graph."

Dr. Thompkins—Must we not always continue to zigzag, as we so rarely find the simillimum?

Dr. Wesselhœft—Certainly, most of our cures will be zigzag cures, but as we become more acquainted with the materia medica we may find the simillimum more often.

Dr. Bell—I really think the antidotal theory is better; it has always seemed more reasonable to me.

Dr. Wesselhœft—My father always accepted Hahnemann's explanation, but thought that the effect of the remedy went with the disease.

Dr. Bell—We are often called upon to explain these things to patients, and the antidotal theory can always be understood by them. Or such an explanation as this, I have seen a carpenter drive a round peg into a hole, and then drive it out again by a similar peg.

Dr. Wesselhœft—The selection of the remedy must always be "similia similibus," but the action of the remedy is "contraria contrariis."

Dr. Thompkins—I recall a case of chronic diarrhœa in which the simillimum was evidently found. The patient, a middle-aged woman, for three years had had an early morning stool driving her out of bed, faintness at stomach, ten to eleven A. M., soreness of the abdomen on standing or walking, especially after a stool. She would have several stools daily. I gave her Sulph.$^{80}$. She had no early morning stool the next day, and since then her stools have been regular and her health perfect. That was six weeks ago.

Dr. Wesselhœft—Has any cutaneous expression appeared?

Dr. Thompkins—Not to my knowledge. I have seen her twice, and I found that from a child she had been accustomed to take crude Sulphur. Once when she had the small-pox her father gave her crude Sulphur in whisky, and she has been taking it all along until a few weeks before the diarrhœa began to trouble her. Now it is a question whether this condition was caused by the crude Sulphur and the potency cured it, or whether the potency made a cure where the crude substance had not prevented the symptoms from coming.

Dr. Wesselhœft—I think we all have such things happen. I have been treating a case of chronic rheumatism with much deformity, with ulcers on the legs, in a neighboring city, through another physician. There is also a nightly diarrhœa, sometimes in the morning. Thuja relieved very much. Then a cough came on some time later for which I sent Puls. The cough became better, the rheumatism worse, but she has had no loose stool since the Puls. She has had this diarrhœa for several years, always after midnight. I do not exactly see why the Puls. did it, although it was a nightly diarrhœa, and I have written for a full account of the diarrhœic symptoms.

I think such cures as the one Dr. Thompkins has related is one of the reasons why the older strict homœopaths are, the more enthusiastic they are. You never see an elderly Hahnemannian without being impressed by his enthusiasm over his cures. He will be much more enthusiastic than the younger men.

Dr. Jameson—The allopaths and mongrel-homœopaths get sadder and sadder the older they get; they become more skeptical.

Dr. Bell—In regard to foot-note 29, which has just been read, this is where they ascribe alternation to Hahnemann, but it is when the symptoms alternate.

Dr. Wesselhœft—Like the alternation of Rhus and Bry. in typhoid fever, he meant when the symptoms change, and Hahnemann by his alternation in such cases meant a dose of the thirtieth this morning of Bry., and then, perhaps, to-morrow night, if the symptoms required, a dose of Rhus$^{30}$, and then, perhaps, in a day or two, the Bryonia symptoms would come up again; but these mongrel homœopaths have been giving Rhus and Bry. in alternation every one to two hours in typhoid ever since. I do not think that now we have to do such things, as we have more remedies. Hahnemann would not do it now, it would not be necessary. I do not recollect a case in which the symptoms alternated regularly. I think we will stop here. The next meeting will be the last for the summer, as Dr. Bell is going away soon, and when he comes back I shall go. So after the next meeting we will adjourn until next fall, when we will finish the *Organon.*

Adjourned to May 19th.

Regular meeting of the Organon Society, held May 19th.

Dr. Wesselhœft read, beginning at the fifty-second section.

Section 53 :

Dr. Wesselhœft—The following illustration is, perhaps, not particularly apropos to this paragraph, but I wish to mention it to show that we must know all of the ailments and conditions of our patients in order to make a thorough cure.

I have been treating a very healthy young lady, whose only difficulty was a trouble with her stomach, and early, profuse menstruation. This dyspepsia would come on periodically, and was characterized by pressure in the stomach with belching of gas, great depression of mind, and intolerance of her condition. One day she came hobbling into my office, and I asked her why she limped. She said her old corn was bothering her again. I looked at the corn (for this was the first time I had heard of it) and found that it was a large, horny growth on the outside of the little toe of the left foot. It was elevated half an inch and looked like a horn with two corn cores. There was a line of inflammation about it which was exquisitely sensitive to pressure on the corn. It seems that all this time she had been going at intervals to her corn-doctor. He would apply iodine and other such things, and, as the corn would improve, the condition of her stomach would get worse. Then she would come to me and I would patch up her stomach and the corn would get worse, but there was no cure of either condition. I told her there must be no more visits to the corn-doctor. There was exquisite tenderness and soreness, which "went all over her" from pressure on the corn, and I had her wear moccasins and such things so that there would be no pressure on it, but there was no change. Three weeks ago I gave her a dose of Sep.$^{cm}$, and she came in to-day to tell me that the corn was dead. It was all shriveled into two little black nodules and could bear any amount of pressure.

It was a curious metastasis. When the corn would get better, in two or three weeks the stomach symptoms would slowly appear, then, as they improved, the corn would gradually get worse, but she never associated the two, or thought they had anything to do with each other. They were two conditions of the same disease, and not two diseases. And now they are both very much better. There were a few stomach symptoms of Sepia, and it also has great sensitiveness to pressure.

Corns may be the result of pressure, but not always. They are very often constitutional, and Hahnemann always mentions the symptoms of the corns in his provings.

Foot-note to section 59.

Dr. Wesselhœft—We ought to make a distinction between nosodes and isopathy. It is a great mistake to give a nosode for the disease from which it is derived on isopathic grounds. We do not give Psorinum for the itch, but it cures the consequences of the itch when indicated by the symptoms.

Dr. Davis—We often give Rhus for Rhus poisoning, do we not?

Dr. Wesselhœft—Yes, we often do, especially if Rhus symptoms are present in a considerable degree. We have had a case at the hospital that was the worst case about of Rhus poisoning I ever saw. Her face was one large scab, with intense itching and burning, great restlessness after midnight. $Rhus^{cm}$ was given with great relief, and she is nearly well now. They were afraid she would scar her face. She would scratch until it was all covered with blood, but her skin is coming out as fair as ever. I always let them scratch. It is perfectly barbarous to tie children's hands to keep them from scratching when they have tinea capitis. I always let them scratch all they please, and it is not true that children scar themselves from scratching.

Dr. Thompkins—I had a case of colic recently with great sensitiveness to pressure, much bloating of the abdomen, and relief from hot, wet clothes.

It was all very quickly relieved by Nux moschata. The next morning, in cautioning the patient not to use spices, she said that nutmeg was her usual spice, and she had eaten of it right along. Yet she was cured by Nux moschata.

Dr. Wesselhœft—Dr. Hering had a case once of chronic headaches that were often relieved by Nux moschata, but never cured. One day he found that the patient was in the habit of eating daily a custard thickly sprinkled with nutmeg. He stopped the nutmeg and there was no return of the headache. I think that Dr. Thompkins' case was a perfectly homœopathic cure, but we must not expect to cure every case of poisoning with the potentized poison.

Section 58.

Dr. Bell—I had a patient recently who had an attack of sciatica two weeks before she came to me. For this Morphine had been given by an allopathic physician. There was no headache or any pain the next day, but a cough developed, for which she came to me. I prescribed for the cough. It began to improve, but in a day or two the sciatica began to return. Then Bryonia cured both the cough and the sciatica.

Section 59.

Dr. Scales—Somebody asked me the other day about Antipyrine. He had heard of some case in which the temperature had been reduced and thought it was a wonderful remedy. I told him the fever was simply a result of disturbances in the system of the patient, and if it was suppressed violently so much the worse for the patient.

Dr. Jameson—Dr. Holcombe, of New Orleans, said this was like large doses of Aconite in yellow fever, and it reminded him of putting a large board on top of a chimney; it stops the smoke but the fire goes on just the same.

Dr. Wesselhœft—This being the last meeting, I trust we will come together again next fall to finish the *Organon*, and then we can begin and read it over again. These meetings have been very instructive and enjoyable, and I think we all learn something new each time we read the *Organon*.

Adjourned until fall.

S. A. KIMBALL, *Secretary*.

